# Reply to Dr. Hendricks’ Review: The Pathology of Typhoid Fever

**Published:** 1868-07

**Authors:** Z. C. McElroy

**Affiliations:** Zanesville, Ohio


ARTICLE XXV.

### REPLY TO DR. HENDRICKS' REVIEW: THE PATHOLOGY OF TYPHOID FEVER.

By Z. C. McELROY, M.D., Zanesville, Ohio.

In this reply to Dr. Hendricks' "*Review of Dr. McElroy's hypothesis of the pathology of typhoid fever*," it is proposed to consider the objections of Dr. H. in the spirit which should animate all controversy, to wit, to give due consideration to all objections to any views advanced. As preliminary, it may be proper to state certain fundamental principles, underlying the science and practice of medicine, generally held in the representative medical mind at this time. These assented to, the way is clear for explanations; objected to, that ends the matter, so far as any views in my paper are concerned. Some common understanding on these points is absolutely necessary to an intelligent discussion.

First. What is Disease? "Disease* is not, as it was formerly imagined to be, a special entity, a particular essence, a something vague, intangible, mysterious, but simply a departure from the normal standard, a change in or of a part, brought about by the perverted action of its circulation, innervation, and nutrition; and modified by structure and function. Nearly every disease, whatever its name or site, is essentially an inflammation. Even in what are called the neuroses, or nervous affections, inflammation generally plays a conspicuous part."

* Prof. Gross' "Now and Then," published address, 1867.

Second. How is it to be studied? "As an object of natural history, in the same manner, and upon the same principle precisely as an animal, a plant, or a mineral is viewed and studied, apart from all hypothesis and speculation, as something tangible and existent, not vague and undefined, without form or substance."*

Third. What are the leading organic processes of life? Nutrition and oxidation, supply and waste, assimilation and destruction. The vegetable builds up organic matter from the inorganic elements and forces of nature. Animals oxidize it, or return its elementary constituents in lower states of organization, or their ordinary states in nature, back to the inorganic world.

Fourth. Force is always conserved as organization is ascending in complexity—the organization itself is the correlation of force—until the highest point is reached in the universe—the tissues of man.—[Grove and Tyndall.]

Fifth. Force invariably reäppears as organization is retrograding, either as heat, mechanical motion, sensation, emotion, or mentality; and the sum of all are the exact equivalent of the destructive metamorphosis accomplished.—[Grove and Tyndall.]

Typhoid fever was studied philosophically, in accordance with these fundamental principles, and before Prof. Gross' formulation of them met my eye, and its empirical phenomena, for the benefit of the medical society over whose deliberations the writer has the honor to preside, and an essay written for, and read to, them, and published in the EXAMINER for March, 1868, p. 139.

Dr. Hendricks reviews this essay in the EXAMINER for May, and objects to the pathological views therein, and thinks they are at variance with known facts; and suggests *friction*, to account for the increased temperature always present in the disease.

Applying the laws of force, and the other general principles

* Prof. Gross' "Now and Then," 1867.

laid down here, in getting a philosophic explanation of the increased temperature in typhoid fever, Dr. H. can hardly conclude otherwise than with myself, that they are due to the peroxidation of the tissues, which so steadily disappear during its progress. If the tissues were suitable for the purposes of life, they could not be oxidized without producing mechanical as well as thermal results, to wit, spasms and convulsions, and there would be absent the febrile phenomena. If nutrition and oxidation were proceeding normally, the result would be health, not disease.

Then, why the peroxidation, and increase of temperature, and waste of tissues in typhoid fever? Can it be otherwise than an interference with the final acts of nutrition, *viz.*, the passage of the liquor sanguinis, through minute cell organizations, to the solid tissues, as well as the normal condition of the blood and solids, by the cause or causes of the disease? These causes being some, not well understood, mode or modes of the ordinary forces of the universe, rendering the tissues unfit for the normal purposes of life—in other words, effete? May it not be that, if the quantity of tissue thus interfered with be small, the result is *intermittent fever?* If larger, *remittent fever?* If still larger, *continued, or typhoid fever*, running its course in two or more weeks, and ending in death or recovery? If, on the other hand, the quantity is excessively large, the result—the *cold plague*, or *pernicious fever*—the dynamics of life, as it were, stamped out—no reäction, no peroxidation set up for its removal, but death in the first rigors or stage?

If Dr. H. will think these things over again, perhaps he may change his conclusions as to Dr. McElroy's pathology of typhoid fever; for, unmistakably, the tendency of the general medical mind is toward regarding diseased action as a unity, modified by structure and function of the parts involved, and by many other causes, as age, sex, climate, season of the year, etc., etc.

